# LncRNA-84277 is involved in chronic pain-related depressive behaviors through miR-128-3p/SIRT1 axis in central amygdala

**DOI:** 10.3389/fnmol.2022.920216

**Published:** 2022-07-26

**Authors:** Xiaowei Jiao, Ruiyao Wang, Xiaobao Ding, Binbin Yan, Yuwen Lin, Qiang Liu, Yuqing Wu, Chenghua Zhou

**Affiliations:** ^1^Jiangsu Key Laboratory of New Drug Research and Clinical Pharmacy, Xuzhou Medical University, Xuzhou, China; ^2^Jiangsu Province Key Laboratory of Anesthesiology, National Medical Products Administration (NMPA) Key Laboratory for Research and Evaluation of Narcotic and Psychotropic Drugs, Xuzhou Medical University, Xuzhou, China

**Keywords:** pain, depression, SIRT1, lncRNA-84277, miR-128-3p

## Abstract

Long-term chronic pain can lead to depression. However, the mechanism underlying chronic pain-related depression remains unclear. Sirtuin 1 (SIRT1) is a nicotinamide adenine dinucleotide (NAD^+^)-dependent histone deacetylase (HDAC). Our previous studies have demonstrated that SIRT1 in the central nucleus of the amygdala (CeA) is involved in the development of chronic pain-related depression. In addition, increasing studies have indicated that long non-coding RNAs (lncRNAs) play a vital role in the pathogenesis of pain or depression. However, whether lncRNAs are involved in SIRT1-mediated chronic pain-related depression remains largely unknown. In this study, we identified that a novel lncRNA-84277 in CeA was the upstream molecule to regulate SIRT1 expression. Functionally, lncRNA-84277 overexpression in CeA significantly alleviated the depression-like behaviors in spared nerve injury (SNI)-induced chronic pain rats, whereas lncRNA-84277 knockdown in CeA induced the depression-like behaviors in naïve rats. Mechanically, lncRNA-84277 acted as a competing endogenous RNA (ceRNA) to upregulate SIRT1 expression by competitively sponging miR-128-3p, and therefore improved chronic pain-related depression-like behaviors. Our findings reveal the critical role of lncRNA-84277 in CeA specifically in guarding against chronic pain-related depression *via* a ceRNA mechanism and provide a potential therapeutic target for chronic pain-related depression.

## Introduction

Chronic pain is one of the common nervous system diseases. More and more studies have shown that long-term chronic pain can lead to negative emotions, such as anxiety, depression, and disgust, which can aggravate the feeling of pain and seriously affect the quality of life of patients (Price, 2000; IsHak et al., 2018). Depression is one of the common negative emotions associated with chronic pain, however, to date there are no effective treatments for chronic pain-related depression. Therefore, it is highly essential to explore the underlying mechanisms of chronic pain-related depression.

Long non-coding RNAs (lncRNAs) are a class of RNA molecules longer than 200 nucleotides. LncRNAs themselves do not encode proteins, but regulate gene expression in the form of RNA at the epigenetic, transcriptional and post-transcriptional levels, thus participating in a variety of physiological or pathological processes (Batista and Chang, 2013; Shi et al., 2013; Schmitz et al., 2016; Chi et al., 2019). Recently, a variety of pain-related lncRNAs have been identified in the spinal cord and dorsal root ganglions (DRG) of rats, mice, and humans, which are involved in the occurrence and development of pain by regulating the expression of pain-related genes and neuronal excitability (Jiang et al., 2015; Zhou et al., 2017; Ray et al., 2018). In addition, increasing studies have indicated that lncRNAs play vital roles in the pathogenesis of depression (Huang et al., 2017; Hosseini et al., 2019). Several lncRNAs in the peripheral blood mononuclear cells (PBMCs) have been identified as the potential biomarkers for diagnosis and therapy response in major depressive disorder (MDD) patients (Cui et al., 2016, 2017). Although studies have shown that lncRNAs are involved in the occurrence and development of chronic pain and depression, however, research on the role of lncRNAs in chronic pain-related depression is limited.

Sirtuin 1 (SIRT1), a nicotinamide adenine dinucleotide (NAD^+^)-dependent histone deacetylase (HDAC), has been shown to regulate multiple biological processes including aging, inflammation, autophagy, cancer, and metabolic diseases (Canto and Auwerx, 2009; Knight and Milner, 2012; Nogueiras et al., 2012; Singh and Ubaid, 2020; Wang et al., 2021). Recently, both clinical and animal experiments have shown that SIRT1 is involved in the development of depression (Lo Iacono et al., 2015; Abe-Higuchi et al., 2016; Kim et al., 2016; Luo and Zhang, 2016). Moreover, our previous studies have demonstrated that in the central nucleus of the amygdala (CeA), a key brain area that regulates both pain and emotional behaviors (Veinante et al., 2013; Neugebauer, 2015; Zhu et al., 2019), of chronic pain-induced depression rats SIRT1 expression is decreased significantly, while SIRT1 overexpression alleviates the depression-like behaviors associated with chronic pain, indicating that SIRT1 plays an important role in the formation of chronic pain-induced depression (Zhou et al., 2020; Sun et al., 2021). However, the upstream mechanisms for SIRT1 to regulate chronic pain-related depression is still unclear.

In our previous study, using a rat neuropathic pain model of spared nerve injury (SNI), we performed lncRNA microarray analysis to identify differentially expressed lncRNAs in the CeA tissues. A novel lncRNA-ENSRNOT00000084277 (abbreviated lncRNA-84277), located at chromosome 16 (chr16:71402764-71406403:+), was identified. The present study aimed to evaluate the role of lncRNA-84277 in chronic pain-related depression and to explore its underlying mechanism. We found that lncRNA-84277 in CeA could function as a competing endogenous RNA (ceRNA) by sponging miR-128-3p to regulate SIRT1 expression and SNI-induced depression-like behaviors.

## Materials and methods

### Animals

Healthy male Sprague-Dawley rats (200–220 g) were maintained 4–5 per cage in specific-pathogen free conditions with (24 ± 1)°C, 40–50% relative humidity, 12/12 h light/dark cycles, and provided with food and water *ad libitum*. The animal care and experimental procedures were approved by the Institutional Animal Care and Use Committee of Xuzhou Medical University (approval number: 202012A145).

### Model of chronic neuropathic pain

The model of chronic neuropathic pain was induced *via* SNI as previously reported (Decosterd and Woolf, 2000). Briefly, a 1–2 cm incision was made along the lateral skin of the left thigh to expose the sciatic nerve and its three branches. The sural nerve was reserved, and the tibial nerve and the common peroneal nerve were carefully ligated with chromic gut ligatures (4.0) and transected. The incision was closed with 4.0 silk sutures. Rats in the sham groups underwent the same surgical procedure but without the ligation or transection.

### Von Frey test

Mechanical allodynia was assessed with von Frey filaments according to the up-down method (Chaplan et al., 1994). Briefly, after a 30-min accommodation period, a series of von Frey filaments (North Coast Medical, Morgan Hill, CA, United States) were applied sequentially to the lateral plantar area of the left hind paw of rats. Each filament was perpendicular to the paw and held for 5 s, and a sharp withdrawal of the hind paw was considered as a positive response. The von Frey filament force (g) that produced a 50% positive response was calculated and expressed as mechanical withdrawal threshold (MWT).

### Acetone test

Cold allodynia was evaluated as previously described (Flatters and Bennett, 2004). After a 30-min accommodation period, a drop of acetone was applied to the lateral plantar surface of the ipsilateral hind paw. The responses of the rats were evaluated by the 4-point method: 0, no response; 1, quick withdrawal or flick of the paw; 2, prolonged withdrawal or repeated flicking of the paw; 3, repeated flicking of the paw with licking directed at the ventral side of the paw. The test was repeated for three times at an interval of 5 min, and the threshold of cold pain in rats was represented as the cumulative scores.

### Forced swim test

The forced swim test was performed as we previously described (Zhou et al., 2020). An animal was placed in a transparent plexiglass cylinder (65 cm in height, 30 cm in diameter) filled with water (25 cm in depth) at (25 ± 1)°C. After a 15 min pretest, 24 h later the animal was allowed to swim in the cylinder for 6 min and the immobility time during the last 5 min was recorded. Immobility was defined as the cessation of all active swimming and escaping activities.

### Sucrose preference test

The sucrose preference test was performed as we previously described (Zhou et al., 2020). Briefly, rats were initially trained to adapt to the sucrose solution before the formal test (Day 1, two bottles of 1% sucrose solution; Day 2, one bottle of 1% sucrose solution and one bottle of water). After the adaptation period, the rats were deprived of water for 23 h. The test was conducted when the rats were housed in individual cages and had free access to two bottles containing the sucrose solution and water, respectively. After 1 h, the weights of the consumed sucrose solution and water were recorded. Sucrose preference (%) was calculated as the consumption of sucrose water divided by the total liquid consumption.

### Open field test

Open field test was performed as we described previously (Zhou et al., 2020). The animal was placed in the central area of an open arena (100 × 100 × 50 cm) and allowed to move freely for 5 min. Locomotor activity of the animal was video-recorded and analyzed by an automated video-tracking system (ANY-maze, Stoelting, Kiel, WI, United States).

### Elevated plus-maze test

The elevated plus-maze apparatus was consisted of two open arms and two closed arms separated by a center square platform (10 × 10 cm). Each arm was 10 cm wide and 50 cm long. The maze was positioned 60 cm from the ground, and 40-cm-high black walls surrounded the closed arms. The apparatus was placed in a mildly lit room different from the room where the animals were maintained and handled. The rat was placed in the center platform and allowed to freely explore for 5 min. Between consecutive tests, the apparatus was cleaned with 75% ethanol solution. The total number of visits were automatically scored using a video tracking system (ANY-maze, Stoelting, Kiel, WI, United States).

### Adeno-associated virus vectors and viral injections

The rAAV2/9-EF1a-lncRNA (LOC102552829-201)-bGH polyA-CMV-mCherry-hGH polyA (AAV-lnc) vector and control vector rAAV2/9-CMV-mCherry-WPRE-polyA (AAV-mCherry), the rAAV2/9-U6-shRNA (LOC102552829-201)-U6-shRNA (LOC102552829-201)-CMV-mCherry-SV40 polyA (AAV-lnc shRNA) vector and control vector rAAV2/9-U6-shRNA (scramble)-U6-shRNA (scramble)-CMV-mCherry-SV40 polyA (AAV-mCherry), and the rAAV2/9-hSyn-BFP-pre-Mir128-2-WPRE-hGH polyA (AAV-miR) vector and control vector rAAV2/9-hSyn-BFP-WPRE-hGH polyA (AAV-BFP) were obtained from BrainVTA Co., Ltd. (Wuhan, China). The pAAV2/9-U6-TuD (rno-miR-128-3p)-CMV-EBFP2-WPRE (AAV-miR TuD) vector and control vector pAAV2/9-U6-shRNA (NC2)-CMV-EBFP2-WPRE (AAV-EBFP) were obtained from OBiO Co., Ltd. (Shanghai, China). The AAV2/9-Hsyn-r-Sirt1-3xflag-ZsGreen (AAV-SIRT1) vector and control vector AAV2/9-Hsyn-ZsGreen (AAV-ZsGreen), and the AAV2/9-Hsyn-mir30-r-Sirt1-ZsGreen (AAV-SIRT1 shRNA) vector and control vector AAV2/9-Hsyn-ZsGreen (AAV-ZsGreen) were obtained from Hanbio Co., Ltd. (Shanghai, China). A volume of 0.3–0.6 μl virus (depending on the expression strength and viral titer) was delivered bilaterally into the CeA of rats (anteroposterior, −2.1 mm from the bregma; mediolateral, ±4.5 mm; dorsoventral, −8.6 mm from dura) at a rate of 0.1 μl/min. Viral injection sites were histologically verified by confirming the fluorescence signal in the CeA of brain slices with a fluorescence microscope.

### Western blotting analysis

The frozen CeA tissues were homogenized in ice-cold RIPA lysis buffer containing protease inhibitors and phenylmethylsulfonyl fluoride (Beyotime Biotech, Jiangsu, China). The supernatants were collected after centrifugation at 12,000 *g* for 15 min at 4°C, and the protein concentration was determined using the BCA Protein Assay Kit (Thermo Scientific, Waltham, MA, United States). Equal amounts of protein samples were separated by SDS-polyacrylamide gel electrophoresis (Beyotime Institute of Biotechnology, China) and transferred onto a nitrocellulose membrane. Then, the membrane was incubated with the following primary antibodies at 4°C overnight: anti-SIRT1 (Cell Signaling Technology, Beverly, MA, United States), and anti-β-actin (Bioworld, Louis Park, MN, United States), followed by incubation with the IRDye 800CW second antibody (Li-Cor, Lincoln, NE, United States). The immunoreactive bands were detected using an Infrared Imaging System (Gene Company Limited, Hong Kong, China) and analyzed with ImageJ software.

### Real-time quantitative PCR

Total RNA was isolated from CeA tissues using a TRIzol reagent kit (Invitrogen, Carlsbad, CA, United States) and reversely transcribed into cDNA using a high-capacity cDNA reverse transcription kit (Applied Biosystems, Foster City, CA, United States). Then the relative mRNA levels were measured by real-time PCR using the Roche 480 LightCycler detection system. Each sample was analyzed in triplicate. The primers used in the RT-qPCR assays are given in Supplementary Table 1. The relative mRNA levels of lncRNA-84277 and SIRT1 were normalized to β-actin. The relative mRNA level of miR-128-3p was normalized to U6.

### Dual-luciferase reporter assay

The wild-type (wt) and mutant type (mut) luciferase reporter vectors for lncRNA-84277 or SIRT1 with miR-128-3p binding regions were constructed by Hanbio Co., Ltd. (Shanghai, China). The vectors were co-transfected with miR-128-3p mimics into 293T cells, and luciferase activities were measured 48 h later using a dual-luciferase reporter system (Promega, Madison, WI, United States).

### Fluorescence *in situ* hybridization

Carboxyfluorescein (FAM)-labeled probe targeting to lncRNA-84277 was designed and synthesized by Wuhan Servicebio Technology Co. Ltd. (Wuhan, China). Probe sequence was shown in Supplementary Table 1. For FISH analysis, PC12 cells were fixed in 4% paraformaldehyde for 20 min. After prehybridization, the cells were hybridized with the specific probe at 37°C overnight. Then, cell nuclei were counterstained with DAPI (4′,6-diamidino-2-phenylindole). Images were photographed using a fluorescence microscope (Tokyo, Japan).

### Nuclear and cytoplasmic fractionation

Nuclear and cytosolic fractions were separated using a Cytoplasmic and Nuclear RNA Purification Kit (Norgen Biotek, Thorold, ON, Canada) according to the manufacturer’s instruction. U6 was used as a nuclear control while β-actin was a cytoplasmic control. The expression levels of β-actin, U6, and lncRNA-84277 in the nuclear and cytoplasm were detected by RT-qPCR assays.

### Statistical analysis

The data are presented as the mean ± SEM. Unpaired Student’s *t*-test was used to compare the differences between two groups. One-way analysis of variance (ANOVA) followed by Bonferroni test was applied to compare the differences among multiple groups. A repeated-measures ANOVA was used to compare the differences of mechanical pain. Mann-Whitney *U*-test was used to compare the differences of cold pain between two groups. Kruskal–Wallis test was used to compare the differences of cold pain among multiple groups. The difference was considered statistically significant at *P* < 0.05.

## Results

### Spared nerve injury rats displays significant depression-like behaviors

**FIGURE 1:**
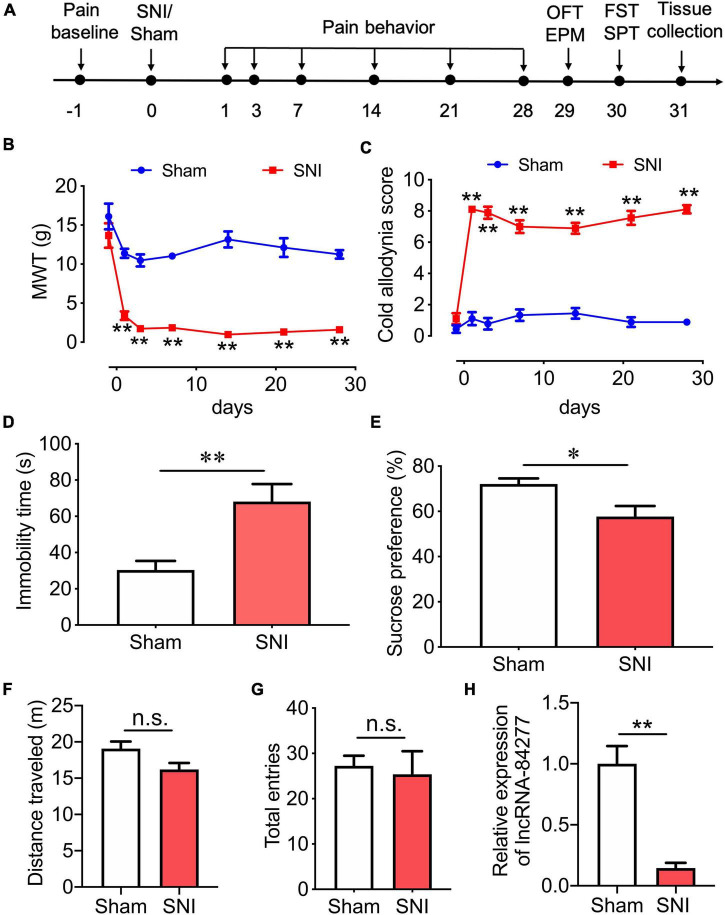
LncRNA-84277 is downregulated in CeA of SNI-induced depression rats. **(A)** Experimental timeline. **(B,C)** Sensory pain was assessed by the mechanical withdrawal threshold (MWT) for mechanical allodynia **(B)** and the cold allodynia score for cold allodynia **(C)** (*n* = 9). **(D,E)** Depression-like behaviors were assessed by the immobility time in the forced swim test **(D)** and sucrose consumption in the sucrose preference test **(E)** (*n* = 8). **(F,G)** Locomotor activity was assessed by the total distance traveled in the open field test **(F)** and the total entries to open arms and closed arms in the elevated plus-maze test **(G)** (*n* = 8). **(H)** The mRNA expression of lncRNA-84277 in the CeA of rats was determined by RT-qPCR (*n* = 8). All data are expressed as the mean ± SEM. *P < 0.05, ^**^P < 0.01. n.s., not significant. OFT, open field test; EPM, elevated plus-maze test; FST, forced swim test; SPT, sucrose preference test.

We used a rat neuropathic pain model of SNI, and the sensory pain was evaluated by Von Frey test and acetone test (Figure 1A). As shown in Figure 1B, compared with sham rats, SNI rats displayed mechanical allodynia with decreased MWT in the ipsilateral hind paw from day 1 to at least day 28 after the SNI surgery. SNI rats also developed cold allodynia, manifested by an increased acetone test score over the same time frame (Figure 1C).

We then determined the depression-like behaviors in SNI rats, using the forced swim test and the sucrose preference test (Figure 1A). We found that, compared with sham rats, SNI rats showed increased immobility time (*P* < 0.01; Figure 1D) and decreased sucrose preference rate (*P* < 0.05; Figure 1E) 30 days after the SNI surgery, suggesting that SNI rats developed depression-like behaviors.

Since motor impairment may influence pain perception and depression-like behaviors, the total distance traveled in the open field test and the total number of arm entries in the elevated plus-maze test were measured as the indicators of locomotor activity (Figure 1A). In the open field test, we found that there was no significant difference in the total distance traveled between sham rats and SNI rats (Figure 1F). In the elevated plus-maze test, the total number of arm entries was not altered in SNI rats when compared with sham rats (Figure 1G). These results indicate that the locomotor activity of the rats was not affected by SNI surgery.

### LncRNA-84277 is downregulated in CeA of spared nerve injury-induced depression rats

In our previous study, we performed lncRNA microarray analysis to identify differentially expressed lncRNAs in the CeA tissues of SNI rats and sham rats, and total 50 novel lncRNAs, including 38 upregulated and 12 downregulated lncRNAs, were identified. To explore the upstream mechanism for SIRT1 to regulate the chronic pain-related depression-like behaviors, we analyzed the differentially expressed lncRNAs, and the novel lncRNA-84277 drew our attention. Further study showed that the expression of lncRNA-84277 in CeA was decreased in SNI rats 31 days after surgery when compared with sham rats (*P* < 0.01; Figure 1H).

### LncRNA-84277 overexpression in CeA improves depression-like behaviors in spared nerve injury rats

**FIGURE 2:**
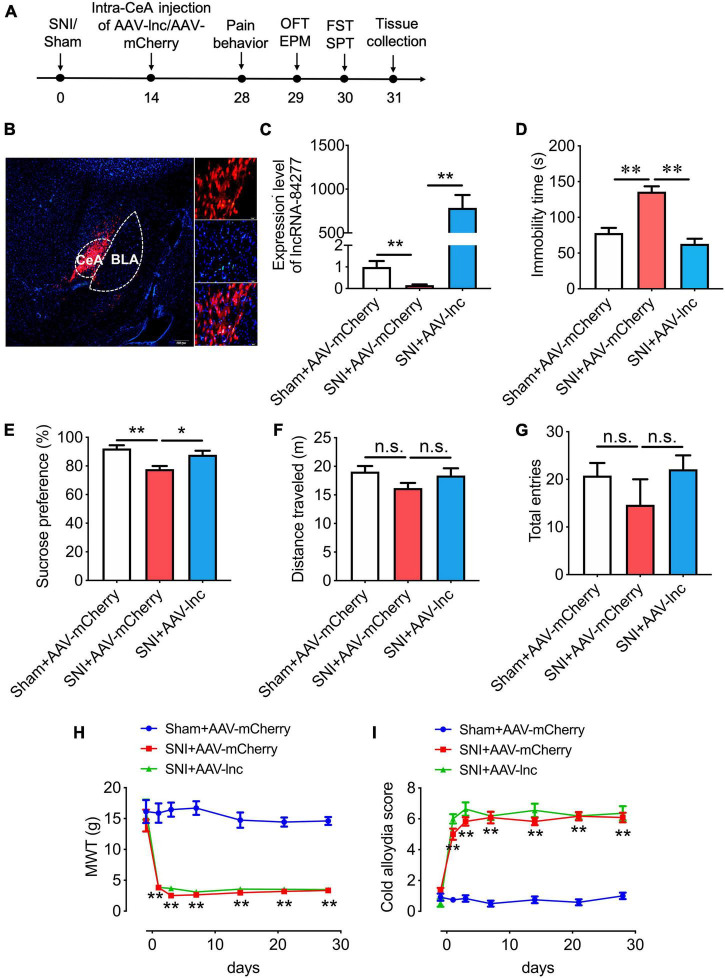
LncRNA-84277 overexpression in CeA improves depression-like behaviors in SNI rats. **(A)** Experimental timeline. **(B)** mCherry fluorescence in CeA after injection of an AAV vector expressing lncRNA-84277 (AAV-lnc). **(C)** RT-qPCR analysis of lncRNA-84277 expression in CeA after injection of AAV-lnc (*n* = 8). **(D,E)** Effect of lncRNA-84277 overexpression on depression-like behaviors in SNI rats assessed by the immobility time in the forced swim test **(D)** and sucrose consumption in the sucrose preference test **(E)** (*n* = 8). **(F,G)** Effect of lncRNA-84277 overexpression on locomotor activity in SNI rats assessed by the total distance traveled in the open field test **(F)** and the total entries to open arms and closed arms in the elevated plus-maze test **(G)** (*n* = 8–9). **(H,I)** Effect of lncRNA-84277 overexpression on sensory pain in SNI rats assessed by the mechanical withdrawal threshold (MWT) for mechanical allodynia **(H)** and the cold allodynia score for cold allodynia **(I)** (*n* = 11–12). All data are expressed as the mean ± SEM. *P < 0.05, ^**^P < 0.01. n.s., not significant. OFT, open field test; EPM, elevated plus-maze test; FST, forced swim test; SPT, sucrose preference test.

Considering that lncRNA-84277 was downregulated in CeA of SNI-induced depression rats, we then investigated whether overexpression of lncRNA-84277 would alter the depression-like behaviors in SNI rats. An AAV vector expressing lncRNA-84277 and mCherry (AAV-lnc) was microinjected into the CeA of SNI rats, and control rats were injected with negative control AAV expressing mCherry only (AAV-mCherry). Then the sensory pain and depression-like behaviors of rats were evaluated 2 weeks after the AAV infusion (Figure 2A). The successful transfection of AAV was verified by the numerous mCherry-positive cells (Figure 2B) and the upregulation of lncRNA-84277 expression in the CeA of SNI rats (*P* < 0.01; Figure 2C). Moreover, lncRNA-84277 overexpression reversed the SNI-induced increase of immobility time in the forced swim test (*P* < 0.01; Figure 2D) and the decrease of sucrose preference rate in the sucrose preference test (*P* < 0.05; Figure 2E). However, the total distance traveled in the open field test (Figure 2F) and the total number of arm entries in the elevated plus-maze test (Figure 2G) did not alter by lncRNA-84277 overexpression. In addition, lncRNA-84277 overexpression had no influence on SNI-induced mechanical allodynia (Figure 2H) and cold allodynia (Figure 2I). These results suggest that the overexpression of lncRNA-84277 in CeA could improve SNI-induced depression-like behaviors.

### LncRNA-84277 knockdown in CeA induces depression-like behaviors in naïve rats

**FIGURE 3:**
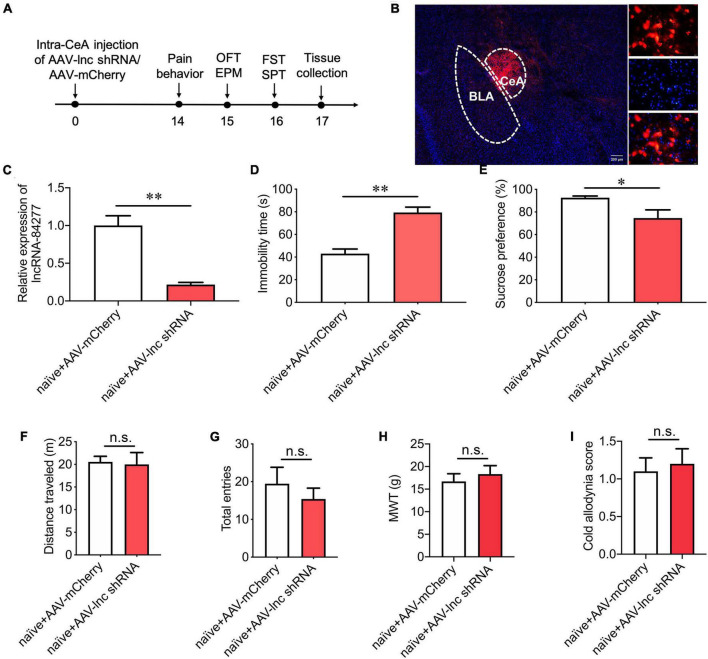
LncRNA-84277 knockdown in CeA induces depression-like behaviors in naïve rats. **(A)** Experimental timeline. **(B)** mCherry fluorescence in CeA after injection of an AAV vector carrying lncRNA-84277-specific shRNA (AAV-lnc shRNA). **(C)** RT-qPCR analysis of lncRNA-84277 expression in CeA after injection of AAV-lnc shRNA (*n* = 8). **(D,E)** Effect of lncRNA-84277 knockdown on depression-like behaviors in naïve rats assessed by the immobility time in the forced swim test **(D)** and sucrose consumption in the sucrose preference test **(E)** (*n* = 8–10). **(F,G)** Effect of lncRNA-84277 knockdown on locomotor activity in naïve rats assessed by the total distance traveled in the open field test **(F)** and the total entries to open arms and closed arms in the elevated plus-maze test **(G)** (*n* = 10–11). **(H,I)** Effect of lncRNA-84277 knockdown on sensory pain in naïve rats assessed by the mechanical withdrawal threshold (MWT) for mechanical allodynia **(H)** and the cold allodynia score for cold allodynia **(I)** (*n* = 10). All data are expressed as the mean ± SEM. *P < 0.05, ^**^P < 0.01. n.s., not significant. OFT, open field test; EPM, elevated plus-maze test; FST, forced swim test; SPT, sucrose preference test.

Next, to further confirm the role of lncRNA-84277 in chronic pain-related depression-like behaviors, an AAV vector carrying lncRNA-84277-specific shRNA (AAV-lnc shRNA) was injected into the CeA of naïve rats, and control rats were injected with a negative control AAV containing scrambled shRNA (AAV-mCherry) (Figure 3A). The successful transfection of AAV-lnc shRNA was verified by the numerous mCherry-positive cells (Figure 3B) and the decreased expression of lncRNA-84277 in the CeA of naïve rats (*P* < 0.01; Figure 3C). Moreover, lncRNA-84277 knockdown induced depression-like behaviors in naïve rats, manifested by the increased immobility time in the forced swim test (*P* < 0.01; Figure 3D) and the decreased sucrose preference rate in the sucrose preference test (*P* < 0.05; Figure 3E). However, the total distance traveled in the open field test (Figure 3F), and the total number of arm entries in the elevated plus-maze test (Figure 3G) did not alter after the knockdown of lncRNA-84277. In addition, lncRNA-84277 knockdown had no influence on the baseline threshold of mechanical pain (Figure 3H) and cold pain (Figure 3I). These results suggest that the knockdown of lncRNA-84277 in CeA is sufficient to induce the depression-like behaviors in the absence of sensory pain in naïve rats.

### LncRNA-84277 acts as a molecular sponge for miR-128-3p involved in chronic pain-related depression

**FIGURE 4:**
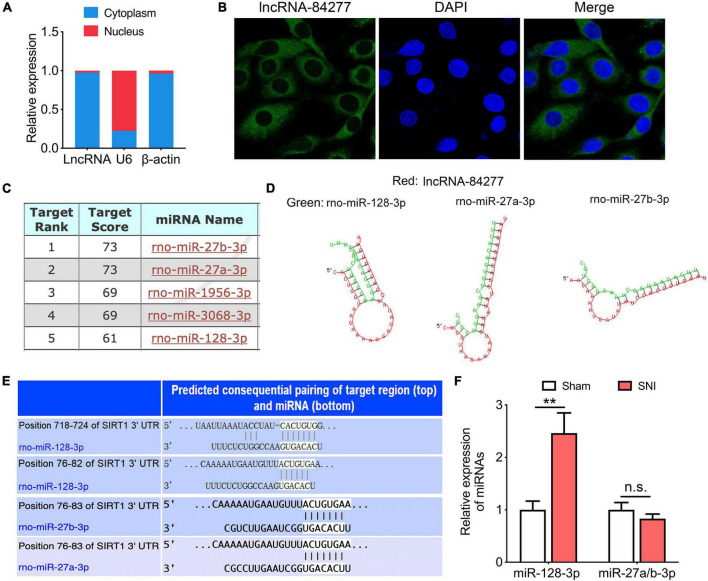
miR-128-3p is a target of lncRNA-84277. **(A)** The subcellular position of lncRNA-84277 in the cytoplasm or nucleus. β-actin and U6 were used as the cytoplasmic and nuclear control, respectively (*n* = 3). **(B)** RNA fluorescence *in situ* hybridization (FISH) for lncRNA-84277 (magnification × 1200). The lncRNA-84277 FISH probe was green and the DAPI nuclear dye was blue. **(C)** The top five miRNAs interacted with lncRNA-84277 predicted by miRDB (http://mirdb.org/). **(D)** The binding sites of the three miRNAs (miR-128-3p, miR-27a-3p, miR-27b-3p) and lncRNA-84277 identified by RNAhybrid (http://bibiserv.cebitec.uni-bielefeld.de/rnahybrid). **(E)** The binding sites of the three miRNAs (miR-128-3p, miR-27a-3p, miR-27b-3p) interacted with SIRT1 identified by TargetScan (http://www.targetscan.org/vert_72/). **(F)** The levels of the above three miRNAs in the CeA of rats were determined by RT-qPCR (*n* = 8). All data are expressed as the mean ± SEM. **P < 0.01. n.s., not significant.

To explore the mechanism for lncRNA-84277 to regulate SIRT1 expression, we observed the cellular localization of lncRNA-84277 using nuclear and cytoplasmic fractionation and FISH experiments. We found that lncRNA-84277 was mainly located in the cytoplasm (Figures 4A,B), suggesting that it may function as miRNA sponge to regulate SIRT1 expression. Therefore, we used the bioinformatic databases (miRDB, RNAhybrid, and TargetScan) to predict the potential miRNAs that interact with both lncRNA-84277 and SIRT1. We found that three of the top five miRNAs binding to lncRNA-84277 (miR-128-3p, miR-27a-3p, and miR-27b-3p, Figures 4C,D) could also bind to the 3’-UTR region of SIRT1 (Figure 4E). We then assessed the expression levels of the three miRNAs in the CeA of SNI-induced depression rats. The results showed that the expression of miR-128-3p increased (*P* < 0.01) while the expression of miR-27a-3p and miR-27b-3p had no significant change (Figure 4F).

**FIGURE 5:**
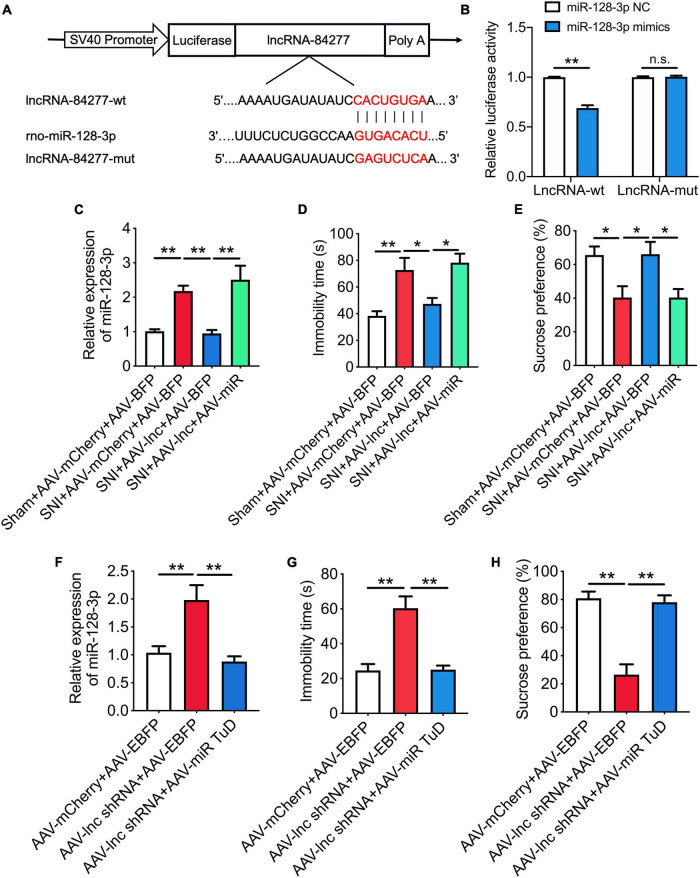
LncRNA-84277 improves depression-like behaviors through inhibiting miR-128-3p. **(A)** The predicted potential binding sites of miR-128-3p to lncRNA-84277 and schematic of wild-type (wt) and mutant-type (mut) of lncRNA-84277. **(B)** Relative luciferase activity of wild type or mutated lncRNA-84277 after co-transfection with miR-128-3p mimics (*n* = 3). **(C)** Effect of lncRNA-84277 overexpression on the level of miR-128-3p and its influence by miR-128-3p overexpression in SNI rats (*n* = 8). **(D,E)** Effect of miR-128-3p overexpression on lncRNA-84277 overexpression-mediated improvement of depression-like behaviors in SNI rats determined by the forced swim test **(D)** and the sucrose preference test **(E)** (*n* = 8). **(F)** Effect of lncRNA-84277 knockdown on the level of miR-128-3p and its influence by miR-128-3p knockdown in naïve rats (*n* = 8). **(G,H)** Effect of miR-128-3p knockdown on lncRNA-84277 knockdown-mediated depression-like behaviors in naïve rats determined by the forced swim test **(G)** and the sucrose preference test **(H)** (*n* = 8). All data are expressed as the mean ± SEM. *P < 0.05, **P < 0.01. n.s., not significant.

Dual-luciferase reporter assay revealed that miR-128-3p mimics reduced the luciferase activity of the lncRNA-84277 wild type reporter (*P* < 0.01) but not the mutated reporter (Figures 5A,B). In addition, overexpression of lncRNA-84277 in CeA reversed the increase of miR-128-3p in SNI rats (*P* < 0.01; Figure 5C), and the improvement of lncRNA-84277 overexpression on the depression-like behaviors in SNI rats was also reversed by miR-128-3p overexpression (Figures 5D,E). Furthermore, knockdown of lncRNA-84277 in CeA induced the increase of miR-128-3p in naïve rats (*P* < 0.01; Figure 5F), and the depression-like behaviors in naïve rats induced by lncRNA-84277 knockdown were reversed by miR-128-3p knockdown (Figures 5G,H). However, the effects of lncRNA-84277 on the locomotor activity and sensory pain were not influenced by miR-128-3p (Supplementary Figures 1A–H). These findings suggest that lncRNA-84277 improves depression-like behaviors through inhibiting miR-128-3p.

### SIRT1 is the downstream target of miR-128-3p

**FIGURE 6:**
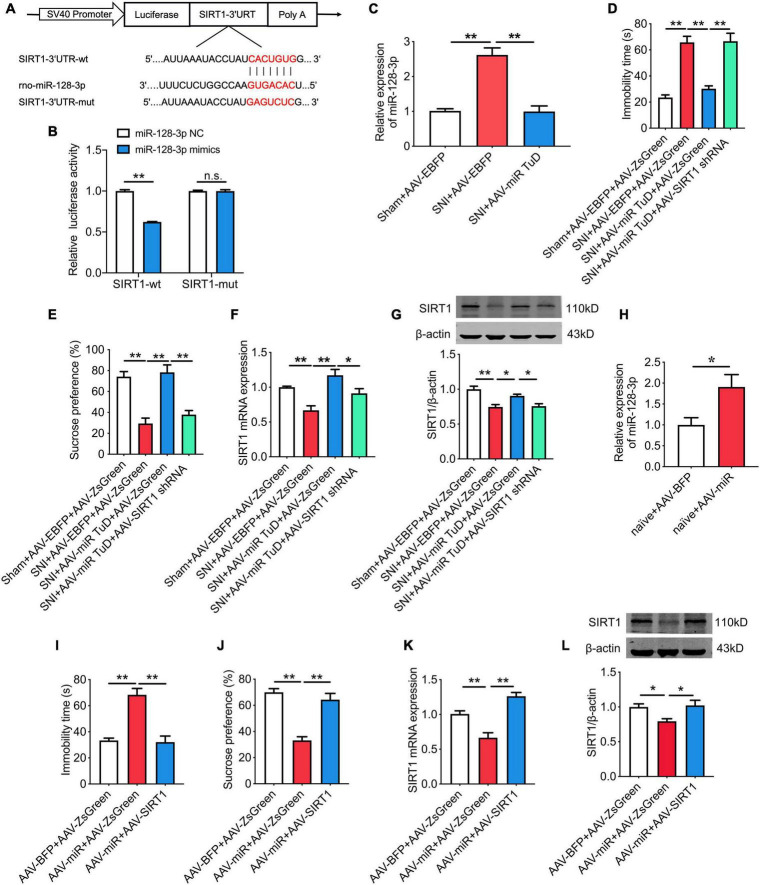
SIRT1 is the downstream target of miR-128-3p and is involved in chronic pain-related depression. **(A)** The predicted potential binding sites of miR-128-3p to SIRT1 and schematic of wild-type (wt) and mutant-type (mut) of SIRT1. **(B)** Relative luciferase activity of wild type or mutated SIRT1 after co-transfection with miR-128-3p mimics (*n* = 3). **(C)** Effect of intra-CeA infusion of AAV-miR-128-3p TuD on the level of miR-128-3p in SNI rats (*n* = 8). **(D,E)** Effect of SIRT1 knockdown on miR-128-3p knockdown-mediated improvement of depression-like behaviors in SNI rats determined by the forced swim test **(D)** and the sucrose preference test **(E)** (*n* = 8). **(F,G)** Effect of SIRT1 knockdown on miR-128-3p knockdown-mediated increase of SIRT1 mRNA **(F)** and protein **(G)** expressions (*n* = 8). **(H)** Effect of intra-CeA infusion of AAV-miR-128-3p on the level of miR-128-3p in naïve rats (*n* = 8). **(I,J)** Effects of SIRT1 overexpression on miR-128-3p overexpression-mediated depression-like behaviors in naïve rats determined by the forced swim test **(I)** and the sucrose preference test **(J)** (*n* = 8). **(K,L)** Effect of SIRT1 overexpression on miR-128-3p overexpression-mediated decrease of SIRT1 mRNA **(K)** and protein **(L)** expressions (*n* = 6–8). All data are expressed as the mean ± SEM. *P < 0.05, **P < 0.01. n.s., not significant.

As shown in Figure 4E, SIRT1 was predicted as a target gene of miR-128-3p. To confirm this finding, we constructed a luciferase reporter vector with the wild type or mutated SIRT1-3’UTR binding site for miR-128-3p (Figure 6A). The results showed that the reduced luciferase activity was observed in SIRT1 wild type reporter (*P* < 0.01) but not in SIRT1 mutated reporter (Figure 6B). Moreover, knockdown of miR-128-3p in CeA by infusion of AAV-miR-128-3p TuD (*P* < 0.01; Figure 6C) relieved the depression-like behaviors and upregulated SIRT1 expression in SNI rats, while these effects were abolished by SIRT1 knockdown (Figures 6D–G). Conversely, overexpression of miR-128-3p in CeA (*P* < 0.05; Figure 6H) induced the depression-like behaviors and decreased SIRT1 levels in naïve rats, which were partially rescued following SIRT1 overexpression (Figures 6I–L). In addition, the effects of miR-128-3p on the locomotor activity and sensory pain were not influenced by SIRT1 (Supplementary Figures 2A–H).

### miR-128-3p/SIRT1 is essential for lncRNA-84277 to regulate chronic pain-related depression

**FIGURE 7:**
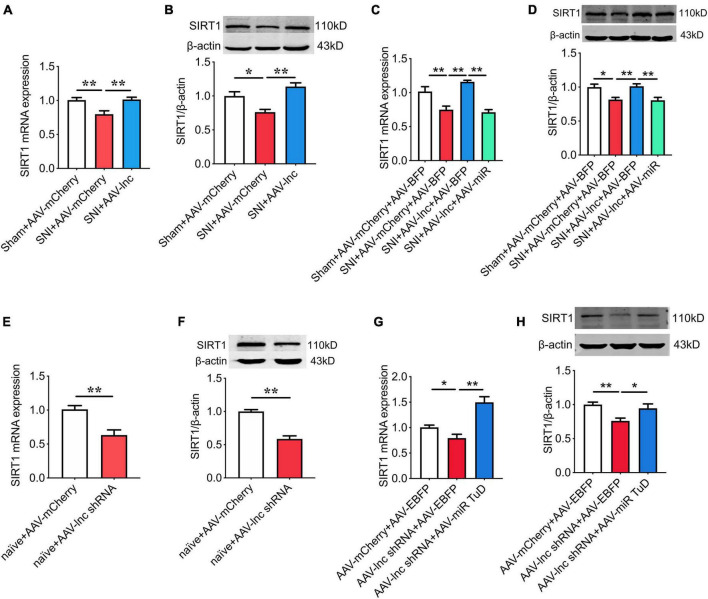
miR-128-3p is essential for lncRNA-84277 to regulate SIRT1 expression. **(A,B)** Effect of lncRNA-84277 overexpression on SIRT1 mRNA **(A)** and protein **(B)** levels in SNI rats. **(C,D)** Effect of miR-128-3p overexpression on lncRNA-84277 overexpression-mediated increase of SIRT1 mRNA **(C)** and protein **(D)** levels in SNI rats. **(E,F)** Effect of lncRNA-84277 knockdown on SIRT1 mRNA **(E)** and protein **(F)** levels in naïve rats. **(G,H)** Effect of miR-128-3p knockdown on lncRNA-84277 knockdown-mediated decrease of SIRT1 mRNA **(G)** and protein **(H)** levels in naïve rats. All data are expressed as the mean ± SEM. *n* = 8 for each group. *P < 0.05, **P < 0.01.

**FIGURE 8:**
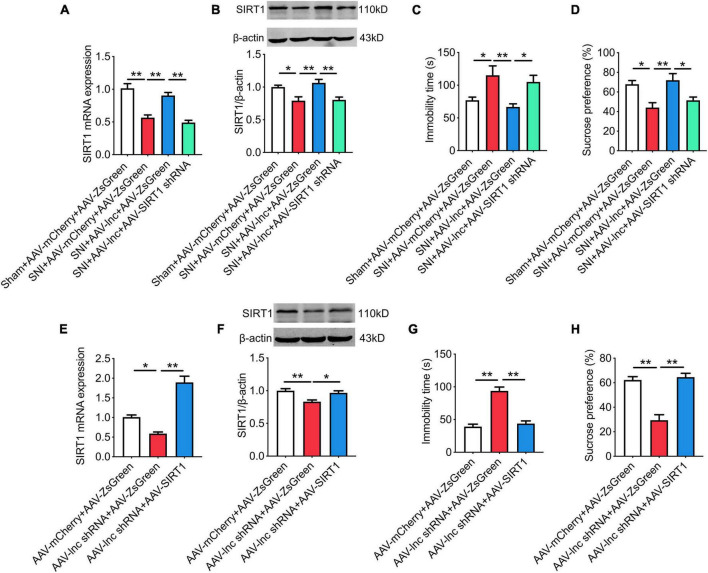
SIRT1 is essential for lncRNA-84277 to regulate chronic pain-related depression. **(A,B)** Effect of SIRT1 knockdown on lncRNA-84277 overexpression-mediated increase of SIRT1 mRNA **(A)** and protein **(B)** levels in SNI rats. **(C,D)** Effect of SIRT1 knockdown on lncRNA-84277 overexpression-mediated improvement of depression-like behaviors in SNI rats determined by the forced swim test **(C)** and the sucrose preference test **(D)**. **(E,F)** Effect of SIRT1 overexpression on lncRNA-84277 knockdown-mediated decrease of SIRT1 mRNA **(E)** and protein **(F)** levels in naïve rats. **(G,H)** Effect of SIRT1 overexpression on lncRNA-84277 knockdown-mediated depression-like behaviors in naïve rats determined by the forced swim test **(G)** and the sucrose preference test **(H)**. All data are expressed as the mean ± SEM. *n* = 8 for each group. *P < 0.05, **P < 0.01.

As shown in Figures 7A,B, Overexpression of lncRNA-84277 in CeA increased SIRT1 expression in SNI rats (*P* < 0.01, *P* < 0.01), which was abrogated by miR-128-3p overexpression (Figures 7C,D). In contrast, SIRT1 expression was inhibited by knockdown of lncRNA-84277 in CeA of naïve rats (*P* < 0.01, *P* < 0.01; Figures 7E,F), and reversed by miR-128-3p knockdown (Figures 7G,H). Similarly, the improvement of the depression-like behaviors and the increased SIRT1 expression in SNI rats caused by lncRNA-84277 overexpression were reversed by SIRT1 knockdown (Figures 8A–D). Conversely, the depression-like behaviors and the decreased SIRT1 expression in naïve rats caused by lncRNA-84277 knockdown were substantially rescued by SIRT1 overexpression (Figures 8E–H). In addition, the effects of lncRNA-84277 on the locomotor activity and sensory pain were not influenced by SIRT1 (Supplementary Figures 3A–H).

**FIGURE 9:**
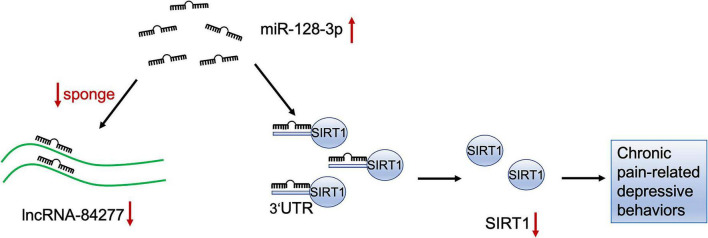
The schematic diagram of the mechanism of lncRNA-84277/miR-128-3p/SIRT1 axis in chronic pain-related depression. LncRNA-84277 downregulation contributes to chronic pain-related depression-like behaviors by increasing the endogenous inhibitory effect of miR-128-3p on SIRT1.

Taken together, these results reveal that lncRNA-84277 serves as a sponge for miR-128-3p to regulate SIRT1 expression and improves SNI-induced depression-like behaviors *via* a ceRNA mechanism (Figure 9).

## Discussion

In this study, we first identified CeA lncRNA-84277 as a significantly downregulated lncRNA in chronic pain-related depression. Second, lncRNA-84277 overexpression in CeA reverses depression-like behaviors in SNI rats, while lncRNA-84277 knockdown in CeA induces depression-like behaviors in naïve rats. Third, lncRNA-84277 improves SNI-induced depression-like behaviors by relieving the suppression of miR-128-3p on SIRT1.

Chronic pain patients are often accompanied by depression, but the underlying pathogenesis is unclear. Recent studies have shown that lncRNAs are involved in the development of chronic pain. For example, in the DRG of chronic constrictive injury (CCI) rats, lncRNA MRAK009713 is implicated in neuropathic pain as a novel positive regulator through regulating the expression and function of the P2X3 receptor (Li et al., 2017). In patients with type 2 diabetes, increased lncRNA NON-RATT021972 is positively associated with neuropathic pain scoring (Yu et al., 2017). In addition, lncRNAs are also reported to participate in the development of depression. Studies have shown that compared with healthy controls, the level of LINC01108 in peripheral blood of patients with depression is significantly increased, while the level of LINC00998 is significantly decreased (Ye et al., 2017). Another study indicates that lncRNA MIR155HG alleviates depression-like behaviors in mice (Huan et al., 2021). However, there are few reports on the roles of lncRNAs in chronic pain-related depression. In our previous work, we have found that CeA SIRT1 plays a vital role in chronic pain-induced depression (Zhou et al., 2020; Sun et al., 2021). Using lncRNA microarray technique, we have identified differentially expressed lncRNAs in the CeA of chronic pain-induced depression rats. Combining lncRNA microarray and bioinformatic prediction, we screened out lncRNA-84277, which had potential regulatory effect on SIRT1. In this study, we showed that lncRNA-84277 was significantly downregulated in the CeA of SNI-induced depression rats. Moreover, overexpression of lncRNA-84277 in CeA improved the depression-like behaviors in SNI rats, while knockdown of lncRNA-84277 in CeA induced the depression-like behaviors in naïve rats. These results indicate that lncRNA-84277 plays a beneficial role in chronic pain-related depression.

As we all known, lncRNAs can regulate gene expressions at multiple levels, such as transcription, post-transcription, and epigenetic regulation (Huang et al., 2012; Grixti and Ayers, 2020). To further explore the mechanism of lncRNA-84277 regulating chronic pain-related depression, we investigated its subcellular localization. FISH and RT-qPCR showed that lncRNA-84277 was mainly expressed in the cytoplasm, which indicated that lncRNA-84277 may be a post-transcriptional regulator. The common mechanism of cytoplasmic lncRNAs is that lncRNAs act as ceRNAs to regulate target mRNA expression by competing for shared miRNAs (Salmena et al., 2011). Hence, we further focused on the potential ceRNA network for lncRNA-84277 to regulate SIRT1 expression. Bioinformatic analysis indicated that three of the top five miRNAs binding with lncRNA-84277 (miR-128-3p, miR-27a-3p, and miR-27b-3p) could simultaneously bind with the 3’-UTR region of SIRT1. Further study showed that the level of miR-128-3p increased significantly in the CeA of SNI-induced depression rats, while the levels of miR-27a-3p and miR-27b-3p had no significant change, suggesting miR-128-3p is involved in the development of chronic pain-related depression. Consistent with our result, it has been reported that in amygdala miR-128-3p may play an important role in depression susceptibility (Roy et al., 2020). Next, dual-luciferase reporter assay was used to determine the relationship of lncRNA-84277 and miR-128-3p, and the results demonstrated that miR-128-3p was a direct target of lncRNA-84277. Moreover, overexpression of lncRNA-84277 in CeA reversed the increase of miR-128-3p in SNI rats, while knockdown of lncRNA-84277 in CeA induced the increase of miR-128-3p in naïve rats. Importantly, the improvement of lncRNA-84277 overexpression on the depression-like behaviors in SNI rats was significantly abolished by miR-128-3p overexpression, while the depression-like behaviors induced by lncRNA-84277 knockdown in naïve rats were markedly reversed by miR-128-3p knockdown. These findings suggest that lncRNA-84277 improves chronic pain-related depression-like behaviors through inhibiting miR-128-3p.

SIRT1, a class-III HDAC, has been found to be involved in chronic pain-associated depression (Zhou et al., 2020; Sun et al., 2021). In the current study, the bioinformatic prediction and dual-luciferase reporter assay demonstrated that SIRT1 was a direct target gene of miR-128-3p. Moreover, miR-128-3p knockdown in CeA markedly relieved the depression-like behaviors and upregulated SIRT1 expression in SNI rats, while miR-128-3p overexpression in CeA induced the depression-like behaviors and decreased SIRT1 levels in naïve rats. Moreover, these effects could be abolished following manipulation of SIRT1 expression. These results suggest that miR-128-3p may be involved in the formation of chronic pain-related depression by targeting SIRT1. Consistent with our results, several studies have reported that miR-128-3p functions in diabetic ulcers, inflammatory responses, liver and kidney injury by targeting SIRT1 (Zhao et al., 2019; Shi et al., 2020; Wu et al., 2020; Ye et al., 2021). Furthermore, our present study also showed that the improvement of the depression-like behaviors and the increased SIRT1 expression in SNI rats caused by lncRNA-84277 overexpression were reversed by miR-128-3p overexpression or SIRT1 knockdown. Conversely, the depression-like behaviors and the decreased SIRT1 expression in naïve rats caused by lncRNA-84277 knockdown were rescued by miR-128-3p knockdown or SIRT1 overexpression. Therefore, we uncovered a new mechanism by which lncRNA-84277 functions as a ceRNA and weakens the endogenous inhibitory effect of miR-128-3p on SIRT1 in the development of chronic pain-related depression.

In addition, there are some limitations in this study. First, both neurons and glial cells have been shown to be involved in chronic pain-related depression (Zhou et al., 2019; Campos et al., 2020). Our previous study has shown that SIRT1 is mainly expressed in GABAergic neurons in CeA (Zhou et al., 2020). However, whether lncRNA-84277 is also mainly expressed in GABAergic neurons or in other cells remains to be further studied. Second, the mechanisms of chronic pain are extremely complex in the brain, which may involve multiple cellular interactions. Therefore, it is essential to explore the role of the interactions between different cells in the development of chronic pain-related depression.

In summary, our results demonstrate that lncRNA-84277/miR-128-3p/SIRT1 axis may act as a new ceRNA regulatory network, participating in the development of chronic pain-related depression. This study is helpful to understand the pathogenesis of chronic pain-related depression and provides potential therapeutic targets for the treatment of chronic pain-related depression.

## Data availability statement

The original contributions presented in this study are included in the article/Supplementary material, further inquiries can be directed to the corresponding authors.

## Ethics statement

The animal study was reviewed and approved by the Institutional Animal Care and Use Committee of Xuzhou Medical University.

## Author contributions

CZ and YW conceived and designed the study. XJ, RW, and XD performed experiments and analyzed the data. BY, YL, and QL helped to perform experiments. CZ and XJ wrote the manuscript. All authors read and approved the final manuscript.

## Conflict of interest

The authors declare that the research was conducted in the absence of any commercial or financial relationships that could be construed as a potential conflict of interest.

## Publisher’s note

All claims expressed in this article are solely those of the authors and do not necessarily represent those of their affiliated organizations, or those of the publisher, the editors and the reviewers. Any product that may be evaluated in this article, or claim that may be made by its manufacturer, is not guaranteed or endorsed by the publisher.

## References

[B1] Abe-Higuchi N. Uchida S. Yamagata H. Higuchi F. Hobara T. Hara K. (2016). Hippocampal sirtuin 1 signaling mediates depression-like behavior. *Biol. Psychiatry* 80 815–826. 10.1016/j.biopsych.2016.01.009 27016384

[B2] Batista P. J. Chang H. Y. (2013). Long noncoding RNAs: cellular address codes in development and disease. *Cell* 152 1298–1307. 10.1016/j.cell.2013.02.012 23498938 PMC3651923

[B3] Campos A. C. P. Antunes G. F. Matsumoto M. Pagano R. L. Martinez R. C. R. (2020). Neuroinflammation, pain and depression: an overview of the main findings. *Front. Psychol.* 11:1825. 10.3389/fpsyg.2020.01825 32849076 PMC7412934

[B4] Canto C. Auwerx J. (2009). Caloric restriction, SIRT1 and longevity. *Trends Endocrinol Metab.* 20 325–331. 10.1016/j.tem.2009.03.008 19713122 PMC3627124

[B5] Chaplan S. R. Bach F. W. Pogrel J. W. Chung J. M. Yaksh T. L. (1994). Quantitative assessment of tactile allodynia in the rat paw. *J. Neurosci. Methods* 53 55–63. 10.1016/0165-0270(94)90144-9 7990513

[B6] Chi Y. Wang D. Wang J. Yu W. Yang J. (2019). Long non-coding RNA in the pathogenesis of cancers. *Cells* 8:1015. 10.3390/cells8091015 31480503 PMC6770362

[B7] Cui X. Niu W. Kong L. He M. Jiang K. Chen S. (2017). Long noncoding RNA expression in peripheral blood mononuclear cells and suicide risk in Chinese patients with major depressive disorder. *Brain Behav.* 7:e00711. 10.1002/brb3.711 28638716 PMC5474714

[B8] Cui X. Sun X. Niu W. Kong L. He M. Zhong A. (2016). Long non-coding RNA: potential diagnostic and therapeutic biomarker for major depressive disorder. *Med Sci Monit.* 22 5240–5248. 10.12659/msm.899372 28039689 PMC5221417

[B9] Decosterd I. Woolf C. J. (2000). Spared nerve injury: an animal model of persistent peripheral neuropathic pain. *Pain* 87 149–158. 10.1016/S0304-3959(00)00276-1 10924808

[B10] Flatters S. J. Bennett G. J. (2004). Ethosuximide reverses paclitaxel– and vincristine-induced painful peripheral neuropathy. *Pain* 109 150–161. 10.1016/j.pain.2004.01.029 15082137

[B11] Grixti J. M. Ayers D. (2020). Long noncoding RNAs and their link to cancer. *Noncoding RNA Res.* 5 77–82. 10.1016/j.ncrna.2020.04.003 32490292 PMC7256057

[B12] Hosseini E. Bagheri-Hosseinabadi Z. De Toma I. Jafarisani M. Sadeghi I. (2019). The importance of long non-coding RNAs in neuropsychiatric disorders. *Mol Aspects Med.* 70 127–140. 10.1016/j.mam.2019.07.004 31319085

[B13] Huan Z. Mei Z. Na H. Xinxin M. Yaping W. Ling L. (2021). lncRNA MIR155HG alleviates depression-like behaviors in mice by regulating the miR-155/BDNF Axis. *Neurochem. Res.* 46 935–944. 10.1007/s11064-021-03234-z 33511575

[B14] Huang X. Luo Y. L. Mao Y. S. Ji J. L. (2017). The link between long noncoding RNAs and depression. *Prog. Neuropsychopharmacol. Biol. Psychiatry* 73 73–78. 10.1016/j.pnpbp.2016.06.004 27318257

[B15] Huang Y. Liu N. Wang J. P. Wang Y. Q. Yu X. L. Wang Z. B. (2012). Regulatory long non-coding RNA and its functions. *J. Physiol. Biochem.* 68 611–618. 10.1007/s13105-012-0166-y 22535282 PMC7098196

[B16] IsHak W. W. Wen R. Y. Naghdechi L. Vanle B. Dang J. Knosp M. (2018). Pain and depression: a systematic review. *Harv. Rev. Psychiatry* 26 352–363. 10.1097/HRP.0000000000000198 30407234

[B17] Jiang B. C. Sun W. X. He L. N. Cao D. L. Zhang Z. J. Gao Y. J. (2015). Identification of lncRNA expression profile in the spinal cord of mice following spinal nerve ligation-induced neuropathic pain. *Mol. Pain* 11:43. 10.1186/s12990-015-0047-9 26184882 PMC4504460

[B18] Kim H. D. Hesterman J. Call T. Magazu S. Keeley E. Armenta K. (2016). SIRT1 mediates depression-like behaviors in the nucleus accumbens. *J. Neurosci.* 36 8441–8452. 10.1523/JNEUROSCI.0212-16.2016 27511015 PMC4978803

[B19] Knight J. R. Milner J. (2012). SIRT1, metabolism and cancer. *Curr. Opin. Oncol.* 24 68–75. 10.1097/CCO.0b013e32834d813b 22080944

[B20] Li G. Jiang H. Zheng C. Zhu G. Xu Y. Sheng X. (2017). Long noncoding RNA MRAK009713 is a novel regulator of neuropathic pain in rats. *Pain* 158 2042–2052. 10.1097/j.pain.0000000000001013 28708759

[B21] Lo Iacono L. Visco-Comandini F. Valzania A. Viscomi M. T. Coviello M. Giampa A. (2015). Adversity in childhood and depression: linked through SIRT1. *Transl. Psychiatry* 5:e629. 10.1038/tp.2015.125 26327687 PMC5068813

[B22] Luo X. J. Zhang C. (2016). Down-regulation of SIRT1 gene expression in major depressive disorder. *Am. J. Psychiatry* 173:1046. 10.1176/appi.ajp.2016.16040394 27690561

[B23] Neugebauer V. (2015). Amygdala pain mechanisms. *Handb. Exp. Pharmacol.* 227 261–284. 10.1007/978-3-662-46450-2_13 25846623 PMC4701385

[B24] Nogueiras R. Habegger K. M. Chaudhary N. Finan B. Banks A. S. Dietrich M. O. (2012). Sirtuin 1 and sirtuin 3: physiological modulators of metabolism. *Physiol. Rev.* 92 1479–1514. 10.1152/physrev.00022.2011 22811431 PMC3746174

[B25] Price D. D. (2000). Psychological and neural mechanisms of the affective dimension of pain. *Science* 288 1769–1772. 10.1126/science.288.5472.1769 10846154

[B26] Ray P. Torck A. Quigley L. Wangzhou A. Neiman M. Rao C. (2018). Comparative transcriptome profiling of the human and mouse dorsal root ganglia: an RNA-seq-based resource for pain and sensory neuroscience research. *Pain* 159 1325–1345. 10.1097/j.pain.0000000000001217 29561359 PMC6008200

[B27] Roy B. Dunbar M. Agrawal J. Allen L. Dwivedi Y. (2020). Amygdala-based altered miRNome and epigenetic contribution of miR-128-3p in conferring susceptibility to depression-like behavior via Wnt signaling. *Int. J. Neuropsychopharmacol.* 23 165–177. 10.1093/ijnp/pyz071 32173733 PMC7171932

[B28] Salmena L. Poliseno L. Tay Y. Kats L. Pandolfi P. P. (2011). A ceRNA hypothesis: the rosetta stone of a hidden RNA language? *Cell* 146 353–358. 10.1016/j.cell.2011.07.014 21802130 PMC3235919

[B29] Schmitz S. U. Grote P. Herrmann B. G. (2016). Mechanisms of long noncoding RNA function in development and disease. *Cell Mol. Life Sci.* 73 2491–2509. 10.1007/s00018-016-2174-5 27007508 PMC4894931

[B30] Shi R. Jin Y. Hu W. Lian W. Cao C. Han S. (2020). Exosomes derived from mmu_circ_0000250-modified adipose-derived mesenchymal stem cells promote wound healing in diabetic mice by inducing miR-128-3p/SIRT1-mediated autophagy. *Am. J. Physiol. Cell Physiol.* 318 C848–C856. 10.1152/ajpcell.00041.2020 32159361

[B31] Shi X. Sun M. Liu H. Yao Y. Song Y. (2013). Long non-coding RNAs: a new frontier in the study of human diseases. *Cancer Lett.* 339 159–166. 10.1016/j.canlet.2013.06.013 23791884

[B32] Singh V. Ubaid S. (2020). Role of silent information regulator 1 (SIRT1) in regulating oxidative stress and inflammation. *Inflammation* 43 1589–1598. 10.1007/s10753-020-01242-9 32410071

[B33] Sun Y. M. Shen Y. Huang H. Liu Q. Chen C. Ma L. H. (2021). Downregulated SIRT1 in the CeA is involved in chronic pain-depression comorbidity. *Brain Res. Bull.* 174 339–348. 10.1016/j.brainresbull.2021.07.002 34245841

[B34] Veinante P. Yalcin I. Barrot M. (2013). The amygdala between sensation and affect: a role in pain. *J. Mol. Psychiatry* 1:9. 10.1186/2049-9256-1-9 25408902 PMC4223879

[B35] Wang L. Xu C. Johansen T. Berger S. L. Dou Z. (2021). SIRT1 – a new mammalian substrate of nuclear autophagy. *Autophagy* 17 593–595. 10.1080/15548627.2020.1860541 33292048 PMC8007159

[B36] Wu L. Zhang G. Guo C. Zhao X. Shen D. Yang N. (2020). MiR-128-3p mediates TNF-alpha-induced inflammatory responses by regulating Sirt1 expression in bone marrow mesenchymal stem cells. *Biochem. Biophys. Res. Commun.* 521 98–105. 10.1016/j.bbrc.2019.10.083 31635801

[B37] Ye N. Rao S. Du T. Hu H. Liu Z. Shen Y. (2017). Intergenic variants may predispose to major depression disorder through regulation of long non-coding RNA expression. *Gene* 601 21–26. 10.1016/j.gene.2016.11.041 27940106

[B38] Ye T. Yang X. Liu H. Lv P. Lu H. Jiang K. (2021). Theaflavin protects against oxalate calcium-induced kidney oxidative stress injury via upregulation of SIRT1. *Int. J. Biol. Sci.* 17 1050–1060. 10.7150/ijbs.57160 33867828 PMC8040307

[B39] Yu W. Zhao G. Q. Cao R. J. Zhu Z. H. Li K. (2017). LncRNA NONRATT021972 was associated with neuropathic pain scoring in patients with type 2 diabetes. *Behav. Neurol.* 2017:2941297. 10.1155/2017/2941297 28928602 PMC5591897

[B40] Zhao X. Jin Y. Li L. Xu L. Tang Z. Qi Y. (2019). MicroRNA-128-3p aggravates doxorubicin-induced liver injury by promoting oxidative stress via targeting Sirtuin-1. *Pharmacol. Res.* 146:104276. 10.1016/j.phrs.2019.104276 31112750

[B41] Zhou C. Wu Y. Ding X. Shi N. Cai Y. Pan Z. Z. (2020). SIRT1 decreases emotional pain vulnerability with associated CaMKIIalpha deacetylation in central amygdala. *J. Neurosci.* 40 2332–2342. 10.1523/JNEUROSCI.1259-19.2020 32005763 PMC7083291

[B42] Zhou J. Fan Y. Chen H. (2017). Analyses of long non-coding RNA and mRNA profiles in the spinal cord of rats using RNA sequencing during the progression of neuropathic pain in an SNI model. *RNA Biol.* 14 1810–1826. 10.1080/15476286.2017.1371400 28854101 PMC5731818

[B43] Zhou W. Jin Y. Meng Q. Zhu X. Bai T. Tian Y. (2019). A neural circuit for comorbid depressive symptoms in chronic pain. *Nat. Neurosci.* 22 1649–1658. 10.1038/s41593-019-0468-2 31451801

[B44] Zhu X. Zhou W. Jin Y. Tang H. Cao P. Mao Y. (2019). A central amygdala input to the parafascicular nucleus controls comorbid pain in depression. *Cell Rep.* 29 3847–3858.e5. 10.1016/j.celrep.2019.11.003 31851918 PMC7020652

