# Supplementary material for: LncRNA-84277 is involved in chronic pain-related depressive behaviors through miR-128-3p/SIRT1 axis in central amygdala
Source: Front Mol Neurosci. 2022 Jul 26;15:920216. doi: 10.3389/fnmol.2022.920216 (PMC9362774; doi:10.3389/fnmol.2022.920216)
Supplement: Supplementary file 1 [file Data_Sheet_1.pdf]

Supplementary Information for

# LncRNA-84277 is Involved in Chronic Pain-Related Depressive Behaviors Through miR-128-3p/SIRT1 Axis in Central Amygdala

Xiaowei Jiao[1†], Ruiyao Wang[1†], Xiaobao Ding[1†], Binbin Yan[1], Yuwen Lin[1], Qiang Liu[2], Yuqing Wu[2*] and Chenghua Zhou[1*]

[1]Jiangsu Key Laboratory of New Drug Research and Clinical Pharmacy, Xuzhou Medical University, Xuzhou, China

[2]Jiangsu Province Key Laboratory of Anesthesiology, NMPA Key Laboratory for Research and Evaluation of Narcotic and Psychotropic Drugs, Xuzhou Medical University, Xuzhou, China

***Correspondence:** Chenghua Zhou

chzhou77@xzhmu.edu.cn

Yuqing Wu

xzmcyqwu@163.com

[†]These authors have contributed equally to this work.

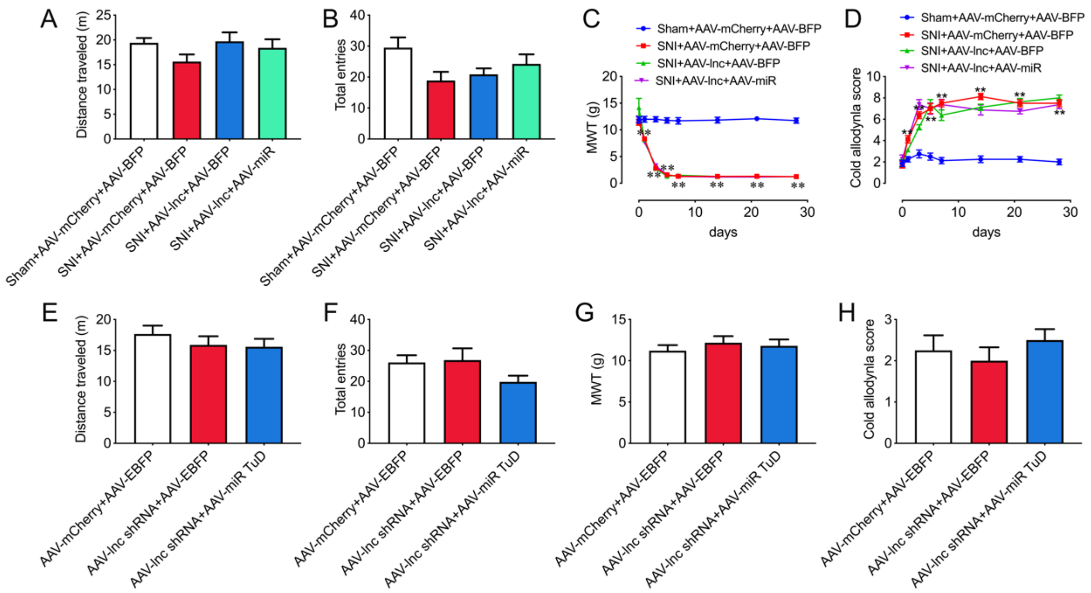


Fig. S1. Effect of lncRNA-84277 on the locomotor activity and sensory pain in rats and the influence of miR-128-3p. (A, B) LncRNA-84277 overexpression did not alter the locomotor activity in SNI rats determined by the open field test (A) and the elevated plus-maze test (B), which was also not influenced by miR-128-3p overexpression (n=8). (C, D) LncRNA-84277 overexpression did not alter the sensory pain in SNI rats, which was also not influenced by miR-128-3p overexpression (n=8). (E, F) LncRNA-84277 knockdown did not alter the locomotor activity in naïve rats determined by the open field test (E) and the elevated plus-maze test (F), which was also not influenced by miR-128-3p knockdown (n=7-8). (G, H) LncRNA-84277 knockdown did not alter the sensory pain in naïve rats, which was also not influenced by miR-128-3p knockdown (n=8). All data are expressed as the mean±SEM. $^{**}P<0.01$ *vs*. Sham+AAV-mCherry+AAV-BFP.

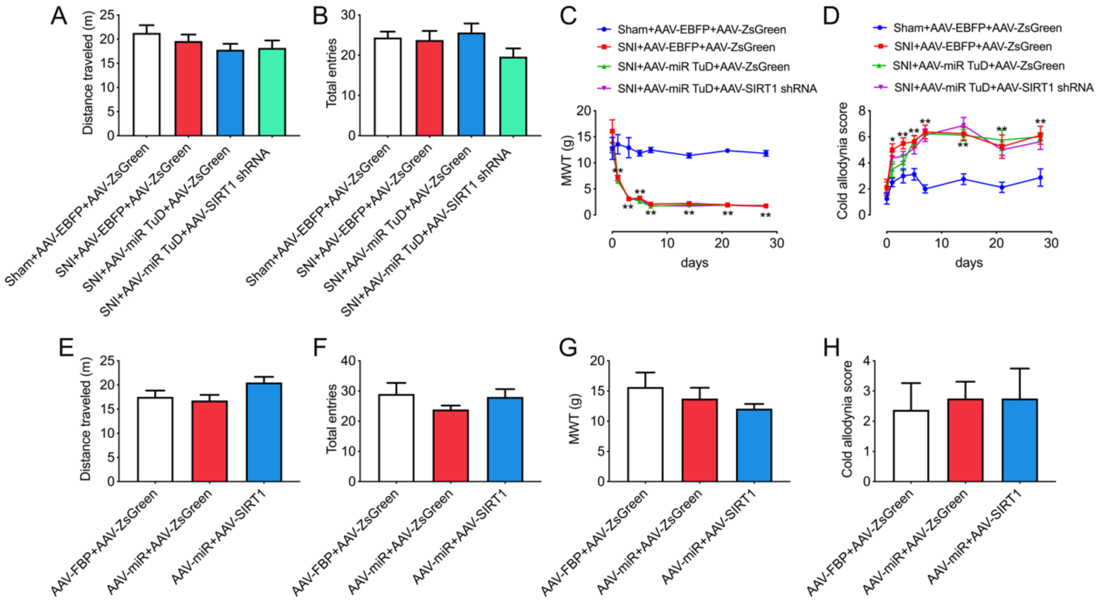


Fig. S2. Effect of miR-128-3p on the locomotor activity and sensory pain in rats and the influence of SIRT1. (A, B) miR-128-3p knockdown did not alter the locomotor activity in SNI rats determined by the open field test (A) and the elevated plus-maze test (B), which was also not influenced by SIRT1 knockdown (n=8). (C, D) miR-128-3p knockdown did not alter the sensory pain in SNI rats, which was also not influenced by SIRT1 knockdown (n=8). (E, F) miR-128-3p overexpression did not alter the locomotor activity in naïve rats determined by the open field test (E) and the elevated plus-maze test (F), which was also not influenced by SIRT1 overexpression (n=7-8). (G, H) miR-128-3p overexpression did not alter the sensory pain in naïve rats, which was also not influenced by SIRT1 overexpression (n=8). All data are expressed as the mean±SEM. $^{**}P<0.01$ *vs*. Sham+AAV-EBFP+AAV-ZsGreen.

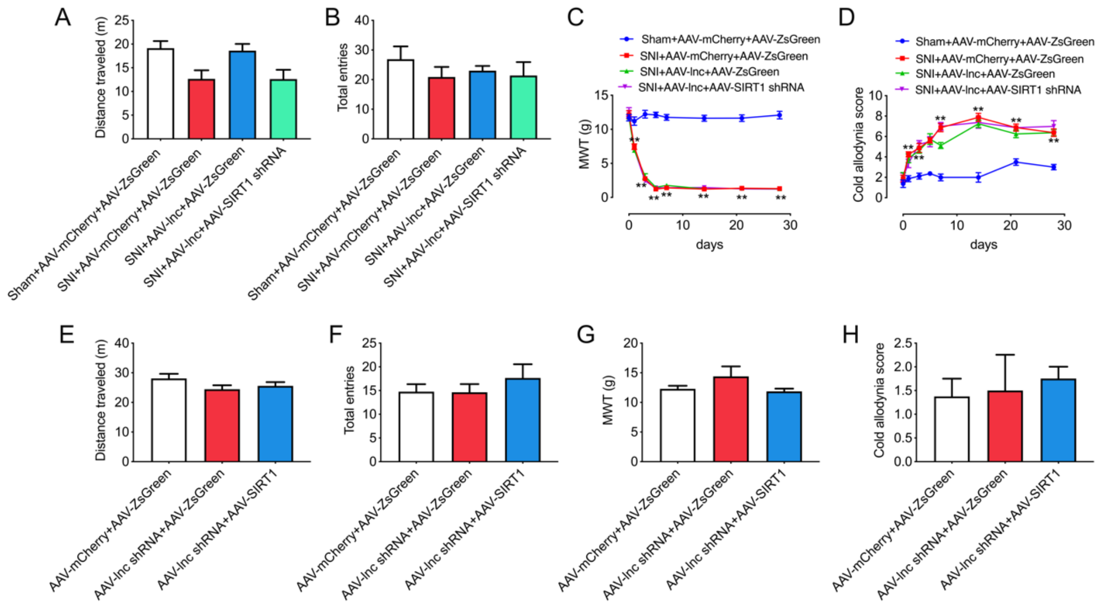

Fig. S3. Effect of lncRNA-84277 on the locomotor activity and sensory pain in rats and the influence of SIRT1. (A, B) LncRNA-84277 overexpression did not alter the locomotor activity in SNI rats determined by the open field test (A) and the elevated plus-maze test (B), which was also not influenced by SIRT1 knockdown. (C, D) LncRNA-84277 overexpression did not alter the sensory pain in SNI rats, which was also not influenced by SIRT1 knockdown. (E, F) LncRNA-84277 knockdown did not alter the locomotor activity in naïve rats determined by the open field test (E) and the elevated plus-maze test (F), which was also not influenced by SIRT1 overexpression. (G, H) LncRNA-84277 knockdown did not alter the sensory pain in naïve rats, which was also not influenced by SIRT1 overexpression. All data are expressed as the mean±SEM. n=8 for each group. ***P*<0.01 *vs*. Sham+AAV-mCherry+AAV-ZsGreen.

Table S1. Primers and RNA sequences used in this study

| Name | Sequence 5'-3' |
|---|---|
| lncRNA-84277-forward | CCTCTGCCCAAACCTTGTCCTTTC |
| lncRNA-84277-reverse | GCAAGCAGAAAGCAGGAAAGCAAC |
| lncRNA-84277-FISH probe | CCAGAAAGGACAAGGTTTGGGCAGAGGTT |
| miR-128-3p-forward | CGCGTCACAGTGAACCGGT |
| miR-128-3p-reverse | AGTGCAGGGTCCGAGGTATT |
| miR-128-3p-RT | GTCGTATCCAGTGCAGGGTCCGAGGTATTCGCAC TGGATACGACAAAGAG |
| U6-forward | AGAGAAGATTAGCATGGCCCCTG |
| U6-reverse | AGTGCAGGGTCCGAGGTATT |
| U6-RT | GTCGTATCCAGTGCAGGGTCCGAGGTATTCGCAC TGGATACGACAAAATA |
| SIRT1-forward | AGGGAACCTCTGCCTCATCT |
| SIRT1-reverse | TGGCATACTCGCCACCTAAC |
| β-actin-forward | CCCATCTATGAGGGTTACGC |
| β-actin-reverse | TTTAATGTCACGCACGATTTC |
